# Is it Safe to Perform Elective Colorectal Surgical Procedures during the COVID-19 Pandemic? A Single Institution Experience with 103 Patients

**DOI:** 10.6061/clinics/2021/e2507

**Published:** 2021-03-15

**Authors:** Lucas Faraco Sobrado, Caio Sergio Rizkallah Nahas, Carlos Frederico Sparapan Marques, Guilherme Cutait de Castro Cotti, Antônio Rocco Imperiale, Pedro Averbach, José Donizeti de Meira, Natally Horvat, Ulysses Ribeiro-Júnior, Ivan Cecconello, Sergio Carlos Nahas

**Affiliations:** I Divisao de Cirurgia Gastrointestinal e Colorretal, Departamento de Gastroenterologia, Faculdade de Medicina FMUSP, Universidade de Sao Paulo, Sao Paulo, SP, BR.; II Departamento de Radiologia, Faculdade de Medicina FMUSP, Universidade de Sao Paulo, Sao Paulo, SP, BR.; III Department of Radiology, Memorial Sloan Kettering Cancer Center, New York, USA.

**Keywords:** COVID-19, Coronavirus infections, Colorectal Surgery, Inflammatory Bowel Diseases, Colorectal Neoplasms

## Abstract

**OBJECTIVES:**

Since the outbreak of the novel coronavirus disease 2019 (COVID-19), all health services worldwide underwent profound changes, leading to the suspension of many elective surgeries. This study aimed to evaluate the safety of elective colorectal surgery during the pandemic.

**METHODS:**

This was a retrospective, cross-sectional, single-center study. Patients who underwent elective colorectal surgery during the COVID-19 pandemic between March 10 and September 9, 2020, were included. Patient data on sex, age, diagnosis, types of procedures, hospital stay, mortality, and severe acute respiratory syndrome coronavirus 2 (SARS-CoV-2) preoperative screening tests were recorded.

**RESULTS:**

A total of 103 colorectal surgical procedures were planned, and 99 were performed. Four surgeries were postponed due to positive preoperative screening for SARS-CoV-2. Surgical procedures were performed for colorectal cancer (n=90) and inflammatory bowel disease (n=9). Laparoscopy was the approach of choice for 43 patients (43.4%), 53 (53.5%) procedures were open, and 3 (3%) procedures were robotic. Five patients developed COVID-19 in the postoperative period, and three of them died in the intensive care unit (n=3/5, 60% mortality). Two other patients died due to surgical complications unrelated to COVID-19 (n=2/94, 2.1% mortality) (*p*<0.01). Hospital stay was longer in patients with SARS-CoV-2 infection than in those without (38.4 *versus* https://doi.org/10.3 days, respectively, *p*<0.01). Of the 99 patients who received surgical care during the pandemic, 94 were safely discharged (95%).

**CONCLUSION:**

Our study demonstrated that elective colorectal surgical procedures may be safely performed during the pandemic; however, preoperative testing should be performed to reduce in-hospital infection rates, since the mortality rate due to SARS-CoV-2 in this setting is particularly high.

## INTRODUCTION

In December 2019, a novel coronavirus—the severe acute respiratory syndrome coronavirus 2 (SARS-CoV-2)—was identified as the cause of a cluster outbreak of pneumonia in Wuhan, China. As a result of the rapid spread, the SARS-CoV-2 infection was first reported in Brazil on February 25, 2020**,** and was declared a pandemic by the World Health Organization on March 11, 2020 (1). All health services established elaborated changes in facing the challenges imposed by the coronavirus disease 2019 (COVID-19) pandemic, having to save resources to deal with critically ill patients infected with the new virus and prevent further in-hospital transmission of patients being treated for other causes (2,3). As a result, many elective surgeries were suspended (4); however, oncological and urgent surgeries were still performed whenever possible (5).

Patients with underlying malignancies and advanced ages are at higher risk for developing severe disease when infected with SARS-CoV-2 (6). Moreover, the inability to receive regular treatments for cancer also increases the risk of cancer-related morbidity, complications, and mortality (7). The management of inflammatory bowel disease (IBD) during the pandemic has become even more challenging due to the risks of balancing immunosuppressant use, timing of surgery, and SARS-CoV-2 infection.

Despite the extensive debate surrounding the best treatment strategies, limited data on surgical results have been published. The surgical treatment for benign disease has been postponed whenever possible, but patients with IBD and cancer often cannot wait. This study aimed to assess the safety of elective colorectal surgery in patients with cancer and IBD during the COVID-19 pandemic.

## METHODS

This was a retrospective, cross-sectional, and single-center study. Data from patients who underwent colorectal surgery were retrospectively reviewed from the surgical database prospectively collected from a single tertiary oncological hospital.

All adult patients of both sexes, without a distinction of ethnicity, who underwent elective colorectal surgery between March 10 and September 9, 2020, were included in this study. No patients were excluded.

During this period, the hospital protocol for elective surgery was in constant adjustment, and until June 1, asymptomatic patients were not routinely tested preoperatively for SARS-CoV-2 infection. After June, every patient scheduled for elective surgery was hospitalized 48 hours prior to the scheduled procedure in private rooms and screened for SARS-CoV-2 infection with polymerase chain reaction (PCR) tests using nasopharyngeal swab and chest computed tomography (CT).

Patients with at least one positive result (chest CT or nasal swab) had their surgery postponed and were treated according to their symptoms and severity of the disease. If hospitalization was required, they were referred to our referral COVID-19 hospital. Patients with both negative results underwent surgery. Postoperative SARS-CoV-2 test was not routinely performed unless acute infection was suspected.

Patient data on sex, age, indication of surgery, diagnosis, types of procedures, hospital stay, mortality, and SARS-CoV-2 screen test results were recorded. Hospital stay was defined as the time elapsed from hospitalization to discharge. After the surgical procedure, patients were clinically monitored during the entire postoperative hospital stay, and if SARS-CoV-2 infection was suspected, chest CT was performed and nasal swab PCR were collected.

During the hospital stay, many precautions were taken to minimize contamination among staff and patients, such as the compulsory use of surgical masks by both patients and staff, minimization of in-person hospital visits, promotion of telemedicine for follow-up whenever possible, and expeditious transfer of infected patients to our referral COVID-19 hospital.

Continuous data are presented as means and standard deviation. Statistical analysis was performed with Student’s t-test to compare hospital stay, and chi-square test was used to compare mortality between patients with and without SARS-CoV-2 infection. The analyses were performed using IBM-SPSS software for Windows version 20.0 (IBM Corp., Armonk, NY, USA).

## RESULTS

During the study period, 103 colorectal surgeries were planned, and 99 colorectal surgical procedures were performed. Four surgeries were postponed due to positive results of preoperative screening for SARS-CoV-2: one patient had positive nasal swab, two patients had chest CT findings suggestive of SARS-CoV-2 infection, and one patient had both tests positive. Patient demographics and postoperative results are summarized in Table 1.

**Figure 1:**
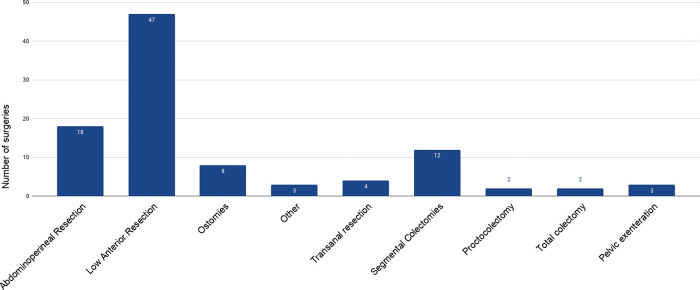
Description of elective colorectal surgical procedures at our hospital during the COVID-19 pandemic.

The most commonly performed surgical procedures in this period were low anterior resection (n=47) and abdominoperineal resection (n=18). Figure 1 shows the surgical procedures performed at our hospital within this period. Other surgeries included the following: hysterectomy and bilateral salpingo-oophorectomy with Hartmann’s procedure reversal in a patient with metastatic disease to the ovaries, right adrenalectomy with colostomy closure in a patient with colorectal cancer metastasis to the adrenal gland, and lateral pelvic lymph node dissection in a patient with rectal cancer metastases.

**Figure 2:**
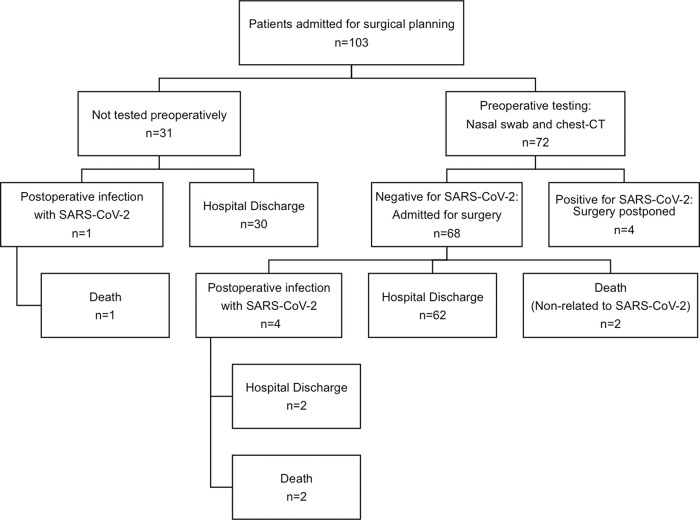
Flowchart demonstrating the results of SARS-CoV-2 testing and surgical outcomes of all patients admitted for elective colorectal surgery during the COVID-19 pandemic.

Figure 2 summarizes the results of SARS-CoV-2 test and surgical outcomes. Preoperative screening for SARS-CoV-2 infection was performed in 72 patients and was positive in 4 (5.6%). All four patients were asymptomatic and had their surgeries postponed.

Of 99 patients who underwent surgery, 5 (5.1%) developed respiratory symptoms during the postoperative period and tested positive for SARS-CoV-2 infection, of which 3 died in the intensive care unit, with a mortality rate of 60% (n=3/5).

The three fatalities due to COVID-19 were described as follows: a 67-year-old man who underwent open low anterior resection for rectal cancer and had a past medical history of diabetes and smoking; a 72-year-old patient with rectal cancer and history of smoking who underwent laparoscopic low anterior resection; and a 72-year-old man with right colon cancer, diabetes, hypertension, and history of smoking who underwent laparoscopic right colectomy. The cause of death was attributed to COVID-19-related complications in all three patients.

Of the patients without SARS-CoV-2 infection, two patients died in the postoperative period: one patient was discharged from the hospital but returned due to acute abdominal pain and was diagnosed with mesenteric ischemia, and the other patient had pulmonary embolism in the early postoperative period (2.1% mortality, n=2/94). The mortality rate was significantly higher in patients infected with SARS-CoV-2 (*p*<0.01). Hospital stay was also significantly longer in patients who developed the infection during the postoperative period (38.4 *versus* 10.4 days; *p*<0.01).

## DISCUSSION

The COVID-19 pandemic is unprecedented and has challenged the medical community to establish fast and effective solutions. At the beginning of the crisis, postponing surgeries and canceling medical appointments seemed to be the most reasonable option (4,8). However, they were soon proven to be inadequate as the pandemic has not subsided, vaccines are still to be developed, and the number of patients waiting for surgical care is increasing.

In Brazil, the pandemic spread rapidly, in part because the authorities denied the severity of the disease, providing a free pass for people who did not want to wear masks and spreading fake news that denied the real mortality of this disease (9). Other factors such as social disparity and high population density in the major cities may have played a role in aggravating the situation. Brazil has reported the second largest number of COVID-19 victims globally, with >4,000,000 cases and 100,000 deaths, the highest numbers being reported in the United States of America (10).

For colorectal surgery, the dilemmas were which patients to prioritize, how to balance the best treatments available, and how to minimize the risks of infection due to in-hospital treatments. Our first decision was to evaluate all medical appointments on an individual basis and decide which patients required in-person evaluation, and whenever possible, the follow-up was conducted through telemedicine.

The treatment for colorectal cancer and IBD was also modified to reduce possible contamination within the hospital.

Rectal cancer treatment was adapted, and some patients were placed in total neoadjuvant therapy protocol with short-course radiotherapy, followed by oral capecitabine, to minimize in-person appointments and avoid daily visits to the hospital, such as in long-course neoadjuvant radiotherapy. Other hospitals in Brazil have adopted similar strategies and oncological treatment (11). Ultimately, the decision for the best treatment strategy should consider individual factors, such as disease status, symptoms, and clinical comorbidities, which should be discussed in a multidisciplinary team, including at least the surgeon and oncologist.

For IBD, patients were administered medications for a longer period at the hospital’s pharmacy and were allowed to self-inject with medications, such as adalimumab, which was previously performed at the infusion center for most patients, since it is considered a high-cost medication in our country (12).

In terms of surgical care, all surgeries for benign diseases (e.g., hemorrhoids, diverticular disease, endometriosis, ostomy closures, and Hartmann’s procedure reversal) were postponed. However, we continued to offer treatment for patients with colorectal cancer who were at risk for progression to unresectable disease and symptomatic IBD despite optimized medical management.

First, we avoided the laparoscopic approach due to the theoretical risk of aerosolization, but we abandoned this practice because of the lack of strong evidence against the known benefits of minimally invasive surgery, such as earlier hospital discharge (13,14). To protect the staff, the number of surgical team members was kept to a minimum, and use of personal protective equipment was made mandatory, which included N-95 masks, waterproof surgical gown, and eye protection. Negative-pressure surgical rooms were unavailable for all elective surgeries and prioritized emergency surgeries in which preoperative testing was not possible.

The results of this protocol included 103 planned colorectal surgeries in a 6-month period. It is interesting to note that the four patients (3.9%) with positive preoperative test were asymptomatic at the time of the examination, making clinical examination alone unreliable in the detection of acute infection. Of these patients, 75% had a positive chest CT, and 50% had a positive swab test, which shows the importance of combining these two tests in the preoperative period.

Postoperative SARS-CoV-2 infection developed in 5 of 99 (5.1%) patients. Although it is possible that their preoperative screening test was performed during the window period of acute infection, and thus a false negative, we considered it more likely that they had in-hospital contamination. To address this, we imposed a stricter visitor policy. At this time, only one visitor was allowed per patient for 1h, and wearing personal protective equipment during the whole period was made mandatory.

The mortality rate of COVID-19 in the postoperative period was considered high, and three of five patients (60%) who developed symptomatic infection died after prolonged hospital stay in the intensive care unit. Nahas et al. have previously published the complicated postoperative course of a few patients who underwent urgent surgery and developed postoperative SARS-CoV-2 infection (15,16). Studies on COVID-19 mortality in the postoperative period are limited, but the general mortality of this infection in patients with cancer is up to 20% (7). It is likely that the mortality rate due to COVID-19 in our series is overestimated because patients were not routinely tested in the postoperative period, just those with a suggestive clinical scenario, which likely led to a selection bias.

We attribute the high mortality found in our study to the disease severity and the profile of patients that attended our hospital, which is a tertiary institution that treats patients with advanced tumors and patients with IBD that have failed initial clinical treatment and frequently have other associated chronic disease, as demonstrated by the ASA classification, in which 28 patients were classified as ASA 3. The profile of surgical procedures was also extremely complex and included 47 low anterior resections, 18 abdominoperineal resections, and three pelvic exenterations. Only 12 of 99 surgical procedures were considered to have minor complexity, which included 8 ostomies and 4 transanal resections. Despite the complexity of surgeries, the clinical profile of our patients and severity of the pandemic, 94 of 99 patients (95%) were safely discharged after elective colorectal surgery during the pandemic.

This observational study provides a panorama of a 6-month period in a tertiary oncological hospital. The main limitations of this study include the lack of preoperative screening tests for 31 patients and a protocol for routine postoperative testing, which may have underestimated the true in-hospital contamination rate but overestimated the lethality of COVID-19 in the postoperative period.

## CONCLUSION

Our study suggests that elective colorectal surgical procedures may be safely performed during the pandemic period, but preoperative testing is paramount, since mortality due to SARS-CoV-2 infection in the postoperative period is particularly high. Hospital stay was also significantly increased in patients infected with the novel virus. Overall, the vast majority of our patients were safely discharged home after elective surgery for IBD and colorectal cancer during the pandemic.

## AUTHOR CONTRIBUTIONS

Sobrado LF was responsible for study conceptualization, manuscript writing and review, and data acquisition. Nahas CSR, Marques CFS, Cotti GCC, and Imperiale AR were responsible for manuscript writing, review and editing. Averbach P, Meira Junior JD, and Horvat N were responsible for data acquisition and literature search. Ribeiro-Junior U, Cecconello I and Nahas SC were responsible for manuscript critical review and study supervision.

## Figures and Tables

**Table 1:** Patient demographics and postoperative results from all patients admitted for elective colorectal surgical procedure.

Total number of patients	n=103
Total number of surgical procedures	n=99
Age, years (mean±SD)	57±14
Sex (male/female)	52/47
American Society of Anesthesiology (ASA)	
Physical Status Classification:	
ASA 1	0
ASA 2	71 (71.7)
ASA 3	28 (28.3)
ASA 4	0
Diagnosis: (n/%)	
Colorectal cancer	90 (90.9)
Inflammatory bowel disease	9 (9.1)
Surgical approach: (n/%)	
Open	53 (53.5)
Laparoscopic	43 (43.4)
Robotic	3 (3)
Hospital stay (days)*	11.7±9.3
Not infected with SARS-CoV-2	10.3±6.7
Postoperative SARS-CoV-2 infection	38.4±13.8
Mortality (n/%)*	
Not infected with SARS-CoV-2	2/94 (2.1%)
Postoperative SARS-CoV-2 infection	3/5 (60%)

* *p*<0.01.

## References

[1] Guan WJ, Ni ZY, Hu Y, Liang WH, Ou CQ, He JX (2020). Clinical Characteristics of Coronavirus Disease 2019 in China. N Engl J Med.

[2] Wexner SD, Cortés-Guiral D, Gilshtein H, Kent I, Reymond MA (2020). COVID-19: impact on colorectal surgery. Colorectal Dis.

[3] Yu J, Ouyang W, Chua MLK, Xie C (2020). SARS-CoV-2 Transmission in Patients With Cancer at a Tertiary Care Hospital in Wuhan, China. JAMA Oncol.

[4] Iacobucci G (2020). Covid-19: all non-urgent elective surgery is suspended for at least three months in England. BMJ.

[5] Carrano FM, Foppa C, Carvello M, Spinelli A (2020). With adequate precautions colorectal cancer surgery can be safely continued during COVID-19 pandemic. Br J Surg.

[6] Dai M, Liu D, Liu M, Zhou F, Li G, Chen Z (2020). Patients with Cancer Appear More Vulnerable to SARS-CoV-2: A Multicenter Study during the COVID-19 Outbreak. Cancer Discov.

[7] Yang K, Sheng Y, Huang C, Jin Y, Xiong N, Jiang K (2020). Clinical characteristics, outcomes, and risk factors for mortality in patients with cancer and COVID-19 in Hubei, China: a multicentre, retrospective, cohort study. Lancet Oncol.

[8] Grubic AD, Ayazi S, Zebarjadi J, Tahmasbi H, Ayazi K, Jobe BA (2020). COVID-19 outbreak and surgical practice: The rationale for suspending non-urgent surgeries and role of testing modalities. World J Gastrointest Surg.

[9] The Lancet (2020). COVID-19 in Brazil: “So what?”. Lancet.

[10] Medicine. JHU Cumulative Cases By Days Since 50th Confirmed Case.

[11] Véo CAR, Cadamuro M, Santo GE, Souza RO, Cesar D, Fernandes PHS (2020). Considerations for the management of colorectal cancer during the Covid-19 pandemic. Braz J Oncol.

[12] Queiroz NSF, Barros LL, Azevedo MFC, Oba J, Sobrado CW, Carlos AS (2020). Management of inflammatory bowel disease patients in the COVID-19 pandemic era: a Brazilian tertiary referral center guidance. Clinics (Sao Paulo).

[13] Mintz Y, Arezzo A, Boni L, Baldari L, Cassinotti E, Brodie R (2020). The risk of COVID-19 transmission by laparoscopic smoke may be lower than for laparotomy: a narrative review. Surg Endosc.

[14] Ribeiro SC, Lauletta ALF, Franco BC, Bezerra RLA, Vanni DGBS, Baracat EC (2020). Laparoscopic surgery and coronavirus disease: What do we know now?. Clinics (Sao Paulo).

[15] Nahas SC, Meira-Junior JD, Sobrado LF, Sorbello M, Segatelli V, Abdala E (2020). Intestinal perforation caused by COVID-19. Arq Bras Cir Dig.

[16] Nahas SC, Meira-Junior JD, Nahas CSR, Sobrado LF, Pinto RA, Abdala E (2020). Results of the surgical treatment of colorectal cancer in COVID-19 patients. Surgical Technology International.

